# Supplementary material for: Hollow-Structured Microporous Organic Networks Adsorbents Enabled Specific and Sensitive Identification and Determination of Aflatoxins
Source: Toxins (Basel). 2022 Feb 13;14(2):137. doi: 10.3390/toxins14020137 (PMC8875801; doi:10.3390/toxins14020137)
Supplement: Supplementary file 1 [file toxins-14-00137-s001.zip › toxins-1587970-supplementary.pdf]

Supplementary Materials: Hollow-Structured Microporous Organic Networks Adsorbents Enabled Specific and Sensitive Identification and Determination of Aflatoxins

Lu Yang, Jin Wang, Huan Lv, Xue-Meng Ji, Jing-Min Liu and Shuo Wang

S1. Experimental section

Synthesis of Fe_3O_4 microparticles

The Magnetic Fe_3O_4 microparticles were prepared via a solvothermal reaction [1]. First, a mixture of $FeCl_3 \cdot 6H_2O$ (27 g), anhydrous sodium acetate (57.5 g) were dissolved into glycol (500 mL) and stirred intensely for 60 min. Then, transferred the system into Teflon-lined autoclaves and reacted at 200°C for 8 h. After cooled to room temperature, black magnetic production was collected and washed with ethanol and ultrapure water for five times. Finally, dried it at 60°C for 12 h.
The silica-coated magnetic Fe_3O_4 microspheres were synthesized as reported previously [2]. Briefly, Fe_3O_4 microspheres obtained previously (1.0 g) were added into 200 mL of HCl (0.1 M) with ultrasonication, then washed with ultrapure water for several times. The Fe_3O_4 microspheres were dispersed in a mixture of ultrapure water (80 mL), ethanol (320 mL), and $NH_3 \cdot H_2O$ solution (5.0 mL, 25–28%). Subsequently, TEOS (1 mL) was introduced to the reaction after ultrasonication for 15 min. The solution was conducted for 12 h with mechanical stirring. The resultants were collected with a magnet, washed with ultrapure water and ethanol for five times and dried under vacuum.

Synthesis of Fe_3O_4@SiO_2@UiO-66-NH_2

The Fe_3O_4@SiO_2@UiO-66-NH_2 were obtained through a solvothermal method. Firstly, Fe_3O_4@SiO_2 (150 mg), Zirconium (IV) chloride (300 mg, 1287 μmol) and water (75 μL) were added to 20 mL N, N-dimethylformamide (DMF) and stirred for 15 min. Then, 2-Aminoterephthalic acid (235 mg, 1298 μmol) was added to 10 mL DMF and stirred until entirely soluble. The above system was transferred into Teflon-lined autoclaves and heated at 120 °C for 24 h. Brownish magnetic materials were obtained after cooled to room temperature, and washed with ultrapure water for five times. Finally, dried under vacuum. In addition, UiO-66-NH_2 was prepared parallelly without the addition of Fe_3O_4@SiO_2 for comparison.

Synthesis of Fe_3O_4@UiO-66-NH_2@MON

For the synthesis of Fe_3O_4 @UiO-66-NH_2@MON, Fe_3O_4@UiO-66-NH_2 (200 mg), $(PPh_3)_2PdCl_2$ (3.4 mg, 4.8 μmol), CuI (1.0 mg, 5.2 μmol) were dispersed with toluene (15 mL) and triethylamine (15 mL) in a 100 mL three-necked flask. After the mixture was sonicated for 0.5 h and mechanically stirred at 90 °C for 30 min, tetrakis(4-ethynylphenyl)methane (50 mg, 0.12 mmol) and 1,4-diiodobenzene (80 mg, 0.24 mmol) were added. Then, the system was reacted at 90 °C for 6 h. After cooled to room temperature, the resultant was separated using a magnet, washed five times with dichloromethane and methanol, dried under vacuum. For the synthesized of the hollow MON, the obtained Fe_3O_4 @UiO-66-NH_2@MON was dissolved into HF solution (48~51%), methanol, and water. Caution: the HF solution is highly dangerous and should be extreme care when used (specific gloves and hood). After stirring for 2 h, centrifuged, washed and dried.

S2. Adsorption kinetics

The adsorption capacity of the as prepared materials was investigated by adsorption kinetics and bonding experiment. Typically, a precisely weighed 10 mg Fe_3O_4@MOF@MON was dispersed in 5 mL

AFT standard solutions with the working concentrations, followed by gently shaking for several time to ensure the sufficiently contact of targets with the adsorption materials. Then, the Fe_3O_4@MOF@MON was collected with an external magnet and the supernatant were determined by HPLC analysis. Several important parameters such as the adsorption capacity of matrices (q) and removal percentage of phytochromes (R%) were calculated by the following equations:

$$q=\frac{(C0-Ce)\times 5}{10}$$

$$R(\%)=\frac{C0-Ce}{C0}\times 100\%$$

where C_0 (mg L^{-1}) and Ce (mg L^{-1}) are AFT concentrations before and after HMON treatment respectively; 5 represents the liquid phase volume (mL) and 10 indicates the mass of HMON (mg).

References

1. S.-W. Lv, J.-M. Liu, C.-Y. Li, H. Ma, Z.-H. Wang, N. Zhao, S. Wang, Fabrication of Fe_3O_4@UiO-66-SO_3H core–shell functional adsorbents for highly selective and efficient removal of organic dyes, New J. Chem., *43*, **2019**, 7770–7777.
2. S.-H. Huo, X.-P. Yan, Facile magnetization of metal–organic framework MIL-101 for magnetic solid-phase extraction of polycyclic aromatic hydrocarbons in environmental water samples, Analyst, *137*, **2012**, 3445–3451.
